# Seroprevalence of SARS-CoV-2 antibodies in the community based on participants in the 2020 Korea National Health and Nutrition Examination Survey

**DOI:** 10.4178/epih.e2022028

**Published:** 2022-02-21

**Authors:** Ah-Ra Kim, Dohsik Minn, Su Hwan Kim, Hyeon Nam Do, Byoungguk Kim, Young Sill Choi, Dong-Hyun Kim, Eun-Jee Oh, Kyungwon Oh, Donghyok Kwon, Jun-Wook Kwon, Sung Soon Kim, June-Woo Lee

**Affiliations:** 1 Division of Vaccine Clinical Research, Center for Vaccine Research, National Institute of Infectious Diseases, Cheongju, Korea; 2 Medical Director for Diagnostic Immunology, Seegene Medical Foundation, Seoul, Korea; 3 Division of Pathogen Resource Management, Center for Vaccine Research, National Institute of Infectious Diseases, Cheongju, Korea; 4 Department of Social and Preventive Medicine, Hallym University College of Medicine, Chuncheon, Korea; 5 Department of Laboratory Medicine, Seoul St. Mary’s Hospital, College of Medicine, The Catholic University of Korea, Seoul, Korea; 6 Division of Health and Nutrition Survey and Analysis, Bureau of Chronic Disease Prevention and Control, Korea Disease Control and Prevention Agency, Cheongju, Korea; 7 Division of Public Health Emergency Responses Research, Korea Disease Control and Prevention Agency, Cheongju, Korea; 8 National Institute of Health (NIH), Korea Disease Control and Prevention Agency, Cheongju, Korea; 9 Center for Vaccine Research, National Institute of Infectious Diseases, Korea Disease Control and Prevention Agency, Cheongju, Korea

**Keywords:** COVID-19, SARS-CoV-2, Antibodies, Seroprevalence, South Korea

## Abstract

**OBJECTIVES:**

The Korea National Health and Nutrition Examination Survey (KNHANES) is a nationwide cross-sectional surveillance system that assesses the health and nutritional status of the Korean population. To evaluate the occurrence of severe acute respiratory syndrome coronavirus 2 (SARS-CoV-2) infection in the community, we investigated the prevalence of anti-SARS-CoV-2 antibodies in the sera of KNHANES participants.

**METHODS:**

Subjects were recruited between April 24 and December 12, 2020. In total, 5,284 subjects aged 10-90 years from 17 regions participated. SARS-CoV-2 antibodies were screened using the Elecsys Anti-SARS-CoV-2 assay. Positive samples were verified using 4 different SARS-CoV-2 antibody assays and the plaque reduction neutralizing test. The final seropositivity criteria were a positive screening test and at least 1 positive result from the 5 additional tests.

**RESULTS:**

Almost half (49.2%; 2,600/5,284) of participants were from metropolitan areas, 48.9% were middle-aged (40-69 years), and 20.5% were in their 20s or younger. The seropositivity rate was 0.09% (5/5,284). Three of the 5 antibody-positive subjects had a history of infection, of whom 2 were infected abroad and 1 was infected in a local cluster outbreak.

**CONCLUSIONS:**

The low SARS-CoV-2 antibody seroprevalence in Korea indicates that there have been few coronavirus disease 2019 (COVID-19) cases due to successful COVID-19 management measures (e.g., diagnostic tests for overseas arrivals, national social distancing, and strict quarantine measures). Moreover, asymptomatic infections were uncommon due to active polymerase chain reaction testing. However, hidden infections may exist in the community, requiring the continuation of quarantine and vaccination measures.

## INTRODUCTION

Coronavirus disease 2019 (COVID-19) first emerged in Wuhan, China in December 2019, and then spread rapidly across the world [1]. The World Health Organization (WHO) declared COVID-19 as a global pandemic on March 11, 2020 [2]. By December 31, 2020, a total of 82,629,416 confirmed cases, including 1,813,193 deaths, were reported worldwide [3]. Korea reported the first confirmed case on January 20, 2020, and by December 31, 2020, 60,740 confirmed cases and 900 deaths had occurred [4-6]. In 2020, there were 3 large COVID-19 waves in Korea. The first outbreak occurred in Daegu in February 2020, the second outbreak in metropolitan areas, while the third outbreak occurred sporadically across the nation in December 2020 [6,7].

Severe acute respiratory syndrome coronavirus-2 (SARS-CoV-2), which causes COVID-19, can spread easily in a population. Although the infection manifests with mild to severe symptoms, there is increasing evidence of asymptomatic infections that can result in viral transmission [8]. Hence, the detection of asymptomatic individuals at an early stage is critical for the prevention and control of COVID-19 spread. Korea reported 3 asymptomatic infections out of 28 confirmed cases (10.7%) in the early stages of an outbreak [9]. Moreover, 62.0% of COVID-19 patients (6,350/10,237) were asymptomatic from January 24 to April 9, 2020, and the asymptomatic positivity rate increased upon pre-emptive testing [10]. Therefore, a study was planned to estimate asymptomatic infections in the community and to assess the seroprevalence of SARS-CoV-2 antibodies in the general population.

The Korea National Health and Nutrition Examination Survey (KNHANES) is a nationwide cross-sectional surveillance system that assesses the health and nutritional status of Koreans through health interviews, examinations, and nutrition surveys. The KNHANES is conducted every year, and includes 10,000 individuals aged 1 year and above [11]. However, the blood and urine samples for the health examination are collected from participants aged 10 years and above. In the present study, we evaluated the efficiency of the existing COVID-19 prevention and control policies by studying the seroprevalence of SARS-CoV-2 antibodies using the KNHANES samples.

## MATERIALS AND METHODS

### Study subjects

This study was conducted on the residual serum samples of KNHANES participants recruited between April 24 and December 12, 2020. At that time, the COVID-19 vaccine had not been introduced in Korea. A total of 5,284 participants aged 10-90 years were recruited from 17 regions, which were classified as provinces and metropolitan areas. The samples were collected 3 times, at 2-month intervals: (1) first collection period (April 24 to June 19) - 1,555 samples, (2) second collection period (June 24 to August 13) - 1,440 samples, (3) third collection period (August 14 to October 31) - 1,379 samples, and (4) fourth collection period (November 1 to December 12) - 910 samples.

### Detection of anti-SARS-CoV-2 antibodies

SARS-CoV-2 antibodies were screened using the Elecsys Anti-SARS-CoV-2 assay on the Cobas e801 analyzer (Roche Diagnostics, Mannheim, Germany). This kit detects antibodies against the nucleocapsid protein of SARS-CoV-2 with a specificity and sensitivity of 100% and 99.8%, respectively [12]. Positive samples were further validated using 5 additional assays that detected various antigens using different detection principles to improve the specificity of the test results: the Architect SARS-CoV-2 IgG assay (Abbott Laboratories, Chicago, IL, USA), Access SARS-CoV-2 IgG assay (Beckman Coulter, Pasadena, CA, USA), R-FIND COVID-19 ELISA (SG Medical, Inc., Seoul, Korea), SGTi-flex COVID19 IgM/IgG (Sugentech, Daejeon, Korea) rapid kits and the plaque reduction neutralizing test (PRNT) for neutralizing antibodies. All test kit assays were performed according to the manufacturer’s instructions.

**Figure 1.**
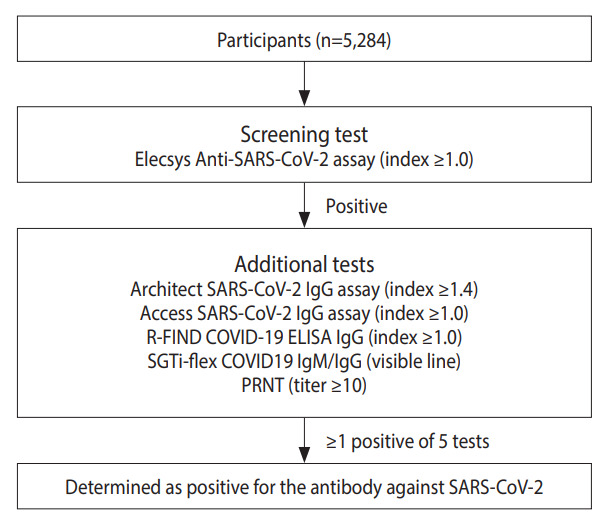
Criteria of severe acute respiratory syndrome coronavirus 2 (SARS-CoV-2) antibody positivity determination. Total 5,284 blood samples of participants were screened using Elecsys Anti-SARS-CoV-2 assay. If the result was positive, five additional antibody tests, including plaque reduction neutralizing test (PRNT), were performed. If one of the five assays was positive, the sample was considered positive for the antibody against SARS-CoV-2.

A sample was ultimately considered positive for SARS-CoV-2 antibodies if both the screening test and at least 1 of the 5 additional tests were positive (Figure 1). All seropositive subjects were checked for their infection and travel history during the pandemic, along with their previous polymerase chain reaction test results.

### Ethics statement

This study was approved by the Institutional Review Board of Korea Disease Control and Prevention Agency (IRB No. 2018-01-03-3C-A).

## RESULTS

The age, sex, and regional distribution of the 5,284 participants are shown in Table 1. The highest number of participants were from the age group of 70 and above (19.0%), followed by those in their 60s and 50s (17.4 and 16.5%, respectively). In contrast, participants in the age ranges of 10-19 years and 20-29 years constituted the smallest groups (9.4 and 11.1%, respectively). The percentages of females and males were 54.8% and 45.2%, respectively. The distribution of study subjects was designed by considering the proportion of population composition by provinces and metropolitan cities. The metropolitan area (Gyeonggi Province and Incheon) had the highest percentage of participants (30.6%), followed by the city of Seoul and Gyeongsang Province (18.6 and 13.0%, respectively). Daegu, the location of the first cluster outbreak, had 3.2% participants.

Thirteen seropositive cases were detected in the screening test, which decreased to 5 after the results of the additional assays were taken into account (Table 2). The assay with the highest similarity to the screening test in performance was the Architect SARS-CoV-2 IgG assay, followed by PRNT. Only 1 sample was positive in the screening test and only 1 additional test, while the rest were positive in 2 or more additional tests.

The number of positive samples in the second, third, and fourth collection periods was 1 (0.07%), 3 (0.22%), and 1 (0.11%), respectively. Among the five seropositive subjects, only 3 (0.06%) had a known history of infection. The confirmed epidemiological information for the 5 seropositive samples is shown in Table 3. Two positive participants were from Seoul, 1 from Gangwon Province, and 2 from Gyeonggi Province. The 2 positive patients from Gyeonggi Province contracted the disease abroad, while the patient in Seoul was infected in a local outbreak. The duration of the antibodies in the seropositive subjects with a history of infection was 12 weeks to 24 weeks.

## DISCUSSION

COVID-19 emerged in December 2019 and spread rapidly worldwide. By the end of December 2020, the United States had the highest number of confirmed cases (19,513,331), while the United Kingdom and Japan had 2,532,601 and 230,304 confirmed cases, respectively [3]. By that time, Korea had reported 60,740 confirmed cases, which was substantially lower than the number in other countries [4,5].

Population-based seroprevalence studies of SARS-CoV-2 have been conducted in several countries. In the United States, the seroprevalence of SARS-CoV-2 antibodies using rapid kits was 1.5% (n=3,330) and 4.3% (n=863) in Santa Clara County and California, respectively [13,14]. In Indiana, where antibodies were tested using chemiluminescence immunoassay (CLIA), the seroprevalence was reported as 2.8% (n=3,658) [15]. More recently, the United States Centers for Disease Control and Prevention conducted a seroprevalence survey comparing the results of 5 serosurveys using random sampling of the general population (n=22,118), and the results were estimated at 14.3% [16]. In England, the COVID-19 seropositivity rate, as reported by Public Health England, was 5.5% using the enzyme-linked immunosorbent assay (ELISA) method (n=7,857) [17]. Turning to the neighboring countries of Korea, China showed a seroprevalence of 1.68% and 0.38% from Wuhan and other places, respectively [18]. In Japan, the Utsunomiya COVID-19 seROprevalence Neighborhood Association study conducted in the city of Utsunomiya reported 0.4% seroprevalence [19]. A nationwide seroprevalence study of SARS-CoV-2 antibodies in Korea was recently published. This study was conducted between late September and early December 2020, and the seroprevalence was 0.39% (16/4,085) [20]. However, our study makes a meaningful contribution in that it analyzed seroprevalence using samples from KNHANES participants obtained over a long period (from April to December 2020). The KNHANES samples were representative of the Korean population, due to the sample design based on the age-specific and region-specific Korean populations.

In this study, the seropositivity of SARS-CoV-2 antibodies in the general population was 0.09% (95% confidence interval, 0.09 to 0.10) (Table 2). This is a very low seroprevalence of SARS-CoV-2 antibodies compared to other countries. This result indicates that the initial testing program was successful in detecting early-stage infections, and the level of hidden infections was maintained at a significantly low level through quarantine control.

The U.S. Food and Drug Administration recommends not using antibody testing for COVID-19 diagnosis because of high false-positive and false-negative rates [21]. Hence, we validated our initial screening test with 5 additional tests to reduce the chances of false-positive or false-negative results. Each additional test used different antigens, methods, and antibody isotypes. The Roche, Abbott, and SG Medical reagents use the nucleocapsid protein as an antigen, the Beckman reagent uses the spike protein, and the Sugentech reagent uses both. As a measurement method, Roche uses electrochemiluminescence immunoassay, Abbott uses chemiluminescence microparticle immunoassay, Beckman uses CLIA, SG Medical uses ELISA, and Sugentech uses lateral flow. The antibody isotypes detected were also diverse, including total antibodies, immunoglobulin (Ig)G, and IgM.

Only 1 case was positive for the antibody in only 2 tests, while the other 4 cases showed positive results in 3 or more tests. Furthermore, neutralizing antibodies against the wild virus were also tested. Eight out of the 13 cases were positive in the screening test only, and since there was a high possibility of these being false-positives, they were excluded from the final positive results (Table 2). All 4 cases that showed positive results in 2 or more binding antibody tests were also positive for neutralizing antibodies. There was no correlation between the binding and neutralizing antibodies, but this relationship was difficult to judge because of the small number of relevant cases.

The KNHANES surveys more than 10,000 people each year. Therefore, we used the KNHANES samples to obtain a large sample size, thereby providing as true a picture of the general population as possible. However, this study has some limitations. First, depending on the time of sampling, there were omissions in the survey area and difficulties in recruiting participants due to the criteria for excluding subjects during the pandemic. For serosurveillance during the COVID-19 pandemic, it is necessary to investigate groups that test routinely and to confirm the history of the diagnosis. To this end, we are conducting additional investigations among military recruits and local communities. Second, the completion date of sample collection (December 12, 2020) might not reflect the third wave of COVID-19 in Korea (late December 2020). Considering the period of antibody production, these findings reflect the prevalence of about 2 weeks before testing. In addition, the formation and duration of antibodies depend on the severity of symptoms [22]. Hence, considering the duration of antibody maintenance, the results might show short-term seroprevalence, rather than a cumulative prevalence. Third, we used only 1 reagent (Roche) in the initial screening test. If more than 1 test had been used for screening, the positivity rate might have been different. Therefore, it is possible that the results were underestimations.

In conclusion, in Korea, the number of COVID-19 cases was low and there were few hidden undiagnosed infections (asymptomatic patients) in the community, due to the thorough 3T strategy (testing, tracing, treatment). Nevertheless, since a small number of undiagnosed infections may exist in the community, it is necessary to maintain quarantine management and active vaccination.

## Figures and Tables

**Table 1.** General information of subjects (n=5,284)

Characteristics	n (%)
Age (yr)	
10-19	499 (9.4)
20-29	588 (11.1)
30-39	612 (11.6)
40-49	792 (15.0)
50-59	871 (16.5)
60-69	919 (17.4)
≥70	1,003 (19.0)
Sex	
Female	2,893 (54.8)
Male	2,391 (45.2)
Region	
Seoul	982 (18.6)
Metropolitan (Gyeonggi, Incheon)	1,618 (30.6)
Gangwon	211 (3.4)
Chungcheong	340 (6.4)
Jeonra	292 (5.5)
Gyeongsang	688 (13.0)
Busan	307 (5.8)
Daegu	171 (3.2)
Gwangju	180 (3.4)
Daejeon	187 (3.5)
Ulsan	94 (1.8)
Sejong	84 (1.6)
Jeju	130 (2.5)
Sample collection period/confirmed cases (n)	
First period (Apr 24-Jun 19)/12,155	1,555 (29.4)
Second period (Jun 24-Aug 13)/14,770	1,440 (27.3)
Third period (Aug 14-Oct 31)/26,511	1,379 (26.1)
Fourth period (Nov 1-Dec 12)/41,736	910 (17.2)

**Table 2.** Seroprevalence of SARS-CoV-2 antibodies^1^

Variables	No. of cases	Screening test	Additional tests				
		Elecsys Anti-SARS-CoV-2 assay	Architect SARS-CoV-2 IgG assay	Access SARS-CoV-2 IgG assay	SG Medical R-FIND COVID-19 ELISA IgG	SGTi-flex COVID19 IgM/IgG	Plaque reduction neutralizing test
Definitely positive for anti-SARS-CoV2	5 (0.09)	5	5	0	0	3	4
Only positive for anti-SARS-CoV2							
In screening test	-	8	0	0	0	0	0
Negative	-	-	0	0	0	0	0
Total	5,284 (100)	13 (0.25)	-	-	-	-	-

SARS-CoV-2, severe acute respiratory syndrome coronavirus 2.

1 If 2 or more test results were positive, a case was considered as definitely positive for the antibody against SARS-CoV-2.

**Table 3.** Characteristics of anti-SARS-CoV-2 antibody test results

Collection period	Age/sex	Roche Elecsys Anti-SARS-CoV-2 assay (ECLIA)		Architect SARS-CoV-2 IgG assay (CLIA)		Access SARS-CoV-2 IgG assay		SG Medical R-FIND COVID-19 ELISA IgG		SGTi-flex COVID-19 IgM/IgG (lateral flow)	Plaque reduction neutralizing test		Result of SARS-CoV-2 PCR test (duration)	Region (domestic or abroad)	Date of sampling (in 2020)	Final result of the antibody against SARS-CoV-2^1^
		Index	Result	Index	Result	Index	Result	Index	Result	Result	Titer	Result				
First	73/F	1.15	+	0.05	-	0.04	-	0.38	-	-	<10.0	-	NA	Chungbuk	May 21	-
	49/F	3.13	+	0.01	-	0.03	-	0.62	-	-	<10.0	-	NA	Incheon	May 29	-
	62/M	1.15	+	0.02	-	0.03	-	0.58	-	-	<10.0	-	NA	Incheon	May 29	-
Second	41/M	136	+	3.57	+	0.36	-	0.88	-	+	10.7	+	NA	Seoul	Jun 30	+
	54/F	1.97	+	0.01	-	0.02	-	0.37	-	-	<10.0	-	NA	Seoul	Jul 02	-
	46/F	1.74	+	0.03	-	0.02	-	0.50	-	-	<10.0	-	NA	Incheon	Jul 08	-
Third	27/M	1.81	+	0.04	-	0.02	-	0.33	-	-	<10.0	-	NA	Gangwon	Sep 17	-
	53/M	70.7	+	2.52	+	0.44	-	0.82	-	+	56.5	+	+(24 wk)	Gyeonggi (abroad)	Sep 26	+
	35/F	50.2	+	1.43	+	0.57	-	0.72	-	-	95.3	+	+(24 wk)	Gyeonggi (abroad)	Sep 26	+
	40/M	10.8	+	0.02	-	0.02	-	0.61	-	-	<10.0	-	NA	Gyeonggi	Oct 08	-
	61/M	17.9	+	2.23	+	0.03	-	0.88	-	-	<10.0	-	NA	Gangwon	Oct 21	+
Fourth	58/F	1.18	+	0.06	-	0.06	-	0.67	-	-	<10.0	-	NA	Gyeongnam	Nov 06	-
	57/F	35.8	+	2.70	+	0.93	-	0.81	-	+	104.4	+	+(12 wk)	Seoul (domestic)	Dec 08	+

SARS-CoV-2, severe acute respiratory syndrome coronavirus 2; ECLIA, electrochemiluminescence immunoassay; CLIA, chemiluminescence immunoassay; NA, not available; F, female; M, male.

1 If 2 or more test results were positive, a case was considered as positive for the antibody against SARS-CoV-2.
